# Moving in and out of Poverty: The Within-Individual Association between Socioeconomic Status and Juvenile Delinquency

**DOI:** 10.1371/journal.pone.0136461

**Published:** 2015-11-17

**Authors:** Roderik Rekker, Dustin Pardini, Loes Keijsers, Susan Branje, Rolf Loeber, Wim Meeus

**Affiliations:** 1 Utrecht University, Utrecht, The Netherlands; 2 University of Pittsburgh, Pittsburgh, Pennsylvania, United States of America; 3 Tilburg University, Tilburg, The Netherlands; University of Bremen, GERMANY

## Abstract

A family’s SES can be changeable over time. This study was the first to investigate if such within-individual changes in family SES are associated with parallel fluctuations in boys’ delinquent behavior from childhood to adolescence. Participants were a community sample of boys and their caregivers (*N* = 503) who were assessed annually for ten consecutive years spanning ages 7–18. Fixed effects models revealed that changes in familial SES were related to changes in delinquency: Youths were more likely to offend during years in which their parents’ SES was lower than during years in which their parents’ SES was higher. Contrary to expectations, we found no evidence that this association was accounted for by families moving to different neighborhoods or by changes in parenting. Since within-individual models provide a stricter test of causality than between-individual models, these findings support claims that impacting familial SES may have a direct effect on youths’ delinquency.

## Introduction

Socioeconomic status (SES) is one of the most well-documented correlates of juvenile delinquency. Many studies have shown that youths from low-SES families are more likely to engage in delinquent behavior than youths from high-SES families [1–3]. In most studies, SES is treated as a static characteristic. However, a family’s SES may in fact be highly changeable [4]. Research shows that, while the child poverty rate in America is 20%, more than half of American youths spend at least one year in poverty before age 18 [5]. Events like job losses or divorces may profoundly change the SES of a family. This raises the question if youths are more likely to offend during years in which their parents’ SES is lower than during years in which their parents’ SES is higher. However, research on such a within-individual association between SES and delinquency and its potential mediators is presently lacking. Hence, the current study was the first to investigate if within-individual changes in family SES (i.e., income, education, occupation, and welfare reliance) are related to concurrent changes in delinquency among children and adolescents between age 7 and 18. Furthermore, this study examined the mediating role of neighborhood quality and parenting.

### Accounts of the Association Between SES and Delinquency

A large body of empirical literature has found that youths from lower SES families are more likely to engage in delinquent behavior [1–3; 6]. Various theoretical accounts propose an explanation for this association. Strain and rational choice accounts argue that delinquency can be a direct result of a family’s low economic resources. According to strain theory (e.g., [7]), youths growing up in poverty may lack the legitimate means to achieve desired social and economic goals. Drawing from rational choice theories (e.g., [8–9]), low-SES youths may have much to gain and little to lose from offending in terms of quality of life and future prospects.

Contrary to this emphasis on economic deprivation, social disorganization theory (e.g., [10–11]) proposes that the relation between SES and delinquency may be mediated by neighborhood quality. Low-SES families may be more likely to live in poor and unstable neighborhoods. Social disorganization theory argues that these neighborhoods facilitate offending due to a lack of social capital and collective supervision. Consistently, research reveals that delinquency is more prevalent in neighborhoods with low levels of affluence and a low residential stability [11–14].

Neighborhood quality is not the only potential mediator of the relation between SES and delinquency. The family stress model (e.g., [15–16]) argues that this relation may alternatively be mediated by parenting. According to control theory (e.g., [17]), a positive parent-child relationship can constitute a social bond that precludes delinquent behavior. A low SES may however create stress for parents, contributing to less positive parent-child relationships [16]. Consistently, research reveals that youths are more likely to offend if their parents experience higher stress levels, know less about their activities, spend less time with them, or fail to consistently reinforce prosocial behavior [6; 18–22]. Some studies have also indicated that family stress and parenting behaviors mediate the association of SES with youths’ externalizing behavior [16; 23] and delinquency [24].

### The Within-individual Association Between SES and Delinquency

Although it is well-documented that *between-individual* differences in family SES are associated with delinquency (e.g., [1]), it is much less clear if *within-individual* changes in family SES are associated with parallel fluctuations in delinquent behavior. In other words, do youths change their delinquent behavior if their family SES changes? Are the same youths more likely to offend during years in which their parents’ SES is lower than during years in which their parents’ SES is higher? Strain and rational choice theories (e.g., [7–8]) suggest that youths may change their delinquent behavior if changes in SES alter their experience of economic deprivation. However, empirical evidence on the within-individual association between SES and delinquency is limited and mostly indirect. For example, a longitudinal study revealed that youths displayed more externalizing behavior in years that their family income was lower than in years that their family income was higher [25]. Furthermore, a quasi-experimental study revealed that youths’ externalizing behavior decreased after their families were lifted from poverty [26]. Likewise, a truly experimental study revealed that a social program that raised families’ SES effectively reduced youths’ externalizing behavior [27]. However, these studies investigated externalizing behavior in general (e.g., anger or stubbornness) rather than (severe) delinquency. One study that focused on delinquency revealed no within-individual association with parental educational level and occupational status [28]. However, this study did not include control variables or measures of economic affluence. As such, the present study was the first comprehensive investigation of how within-individual changes in familial SES are related to concurrent changes in juvenile delinquent behavior.

While little is known about the within-individual association between SES and delinquency, even less is known about its potential mediators. Social disorganization theory (e.g., [10–11]) suggests that youths may change their delinquent behavior if changes in SES lead their family to reside in a new neighborhood. Consistent with this notion, an experimental study revealed that youths’ violent delinquent behavior was reduced by relocating their families to more affluent neighborhoods [29]. The family stress model (e.g., [15–16]) suggests that youths may change their delinquent behavior if changes in SES affect the relation with their parents. A quasi-experimental study identified parenting as a mediator of the association between changes in income and changes in youths’ externalizing behavior [26]. Likewise, longitudinal studies on delinquency revealed that changes in parent-child relationships are related to within-individual changes in offending [28; 30–31]. However, the present study was the first to examine if neighborhood quality and parenting practices mediate the hypothesized within-individual association between SES and delinquency.

### Conflicting Theory

Although several theoretical accounts suggest that youths may change their delinquent behavior as their family SES changes, it should be emphasized that such a within-individual association is controversial from some other perspectives. First, social selection accounts argue that the relation between SES and delinquency is at least partly spurious [32–34]. Parental characteristics like a low IQ or a genetic propensity for criminal behavior may contribute both to their low SES and to their offspring’s delinquency. By definition, changes in SES cannot alter such preexisting risk factors. Second, cultural deviance accounts (e.g., [35]) argue that it is not so much the economic, but rather the cultural component of a low SES that contributes to delinquency. Lower-class youths may be socialized with values and behavioral patterns that contribute to delinquency and preclude functioning in conventional society. It seems implausible that short-term changes in SES could directly affect such cultural orientations. Third, life-course accounts (e.g., [36–37]) argue that the development of delinquency depends on an exposure to risk factors during different ages. This implies that the association between SES and delinquency in late childhood and adolescence could originate from parents’ SES during earlier life-phases. As such, changes in SES may come too late to alter delinquent behavior. This study aimed to contribute to this theoretical debate by assessing the existence of a within-individual association as well as its mediators.

### The Present Study

In sum, the primary aim of this study was to investigate if within-individual changes in family SES are associated with concurrent changes in juvenile delinquency. To provide a reference and facilitate interpretability, we assessed all associations between as well as within individuals. We addressed two research questions. First, is SES related to delinquency? We expected that a lower SES would be related to a higher probability of offending (H1). Second, do neighborhood quality and parenting mediate the relation between SES and delinquency? We expected that a lower neighborhood quality (H2) and less positive parenting practices (H3) would be related to a higher probability of offending. Furthermore, we expected that neighborhood quality (H4) and parenting (H5) mediate the association between SES and delinquency.

## Method

### Participants

We used data from the youngest cohort of the Pittsburgh Youth Study [6]. This study began in 1987 by contacting 1004 boys enrolled in the first grade of Pittsburgh primary schools. Of those contacted, 849 boys and their caretakers agreed to participate in a screening. Based on this screening, 256 boys who scored in the upper 30% on antisocial behavior were selected for a longitudinal follow up, together with 247 boys who were randomly selected from the remaining respondents. This method allowed us to study a large number of delinquent outcomes, while retaining the general population as a reference. The 503 selected boys participated in 10 annual waves. Sample characteristics (displayed in Table 1) indicate that the sample featured a strong representation of low-SES families. Consequently, within-individual changes in SES occurred largely around the poverty line.

**Table 1:** Sample Characteristics.

Variable	Distribution
Age at wave 1	Mean: 7.96 years; Range: 7–10 years
Ethnicity	55.7% African-American
	40.6% European descent
	1.0% Asian
	2.8% Other
Single parent family	36.0% Single parent
Parental education	0.4% 6th Grade or less
	3.0% 7th–9th Grade
	9.5% Some high school
	59.8% High school graduate
	16.3% Some college
	6.6% College graduate
	4.4% Advanced degree
Welfare reliance	50.6% Receiving welfare
Annual household income at wave 1	Mean: $16,450
	10% below $4,606; 10% over $34,000
Inflation adjusted household income at wave 10	Mean: $22,926
	10% below $5,214; 10% over $47,075

### Ethics Statement

Respondents and their caregivers provided written informed consent for their cooperation and for the release of other types of information such as juvenile court records. This informed consent was renewed throughout the study. Specifically, respondents provided written assent until age 18 and then provided consent. The Pittsburgh Youth Study was approved by the Institutional Review Board at the medical school of the University of Pittsburgh.

### Measures

#### Delinquency

Delinquency was assessed using three sources: self-report, primary caretakers, and convictions. Youths reported on their delinquent behavior by completing the *Self-Reported Antisocial Behavior Scale* (SRA) [38], the *Self-Reported Delinquency Scale* (SRD) [39], and the *Youth Self-Report* (YSR) [40]. Because the SRD and YSR were judged to be inappropriate at the earliest ages, youths were administered the SRA at the first three waves. At the fourth wave, the SRA was replaced by the SRD and the YSR. Primary caregivers reported on youths’ delinquency by completing the *Child Behavior Checklist* (CBCL) [41]. Convictions were obtained from official juvenile court records.

Since research generally reveals stronger links with SES for more severe offenses [1; 42], these three sources were combined to create three dichotomous delinquency constructs at each wave: minor delinquency, moderate delinquency, and serious delinquency. Minor delinquency refers to non-violent offences such as stealing a bicycle or skateboard from the street, stealing something worth more than $5, joyriding, purse snatching, dealing in stolen property, or larceny. Moderate delinquency refers to gang fighting or simple assault. Serious delinquency refers to burglary, auto theft, forcible robbery, aggravated assault, rape, or homicide. Each construct was positive if an offense was indicated by either of the sources for the previous year. About half of respondents committed minor delinquency during the course of the study (49.9%), whereas about one third committed moderate delinquency (34.0%) or serious delinquency (31.6%).

#### Family SES

Family SES was operationalized as a composite of educational level, household income, occupation, and being on welfare. We viewed family SES as a formative latent variable, which implies that is is merely a summation of its indicators [43]. Educational level was measured on a scale ranging from 1 (6^th^ grade or less) to 7 (advanced degree). Occupation was measured using the Hollingshead index [44] with scores ranging from 0 (unemployed) to 9 (executive or major professional). For both educational level and occupation, a family’s score reflected the highest score obtained by either the male or the female caretaker. The annual household income was first adjusted for inflation to make scores comparable across waves. Since percentagewise changes in income may be more meaningful than absolute changes, scores were then transformed as ln(income + 1000). The welfare construct was positive if anyone in the household had received public assistance during the previous year. The four indicators of SES displayed a considerable average intercorrelation of .45. Family SES was calculated as the standardized sum of the standardized scores on the indicators. Family SES and other constructs in this study were standardized (across all waves) to create a common metric and facilitate interpretability. For all constructs, we calculated rank-order stabilities (correlations between scores at different waves), to assess how many changes occurred within individuals over time. Importantly, family SES displayed substantial over-time variability with a rank-order stability of .93 from the first to the second year and .65 from the first wave to year 10.

#### Neighborhood quality

For neighborhood quality, we used data from the US census bureau on census tract level. We distinguished between neighborhood affluence and stability. Neighborhood affluence constituted a composite of neighborhoods’ median household annual income, proportion of families below poverty level, proportion of unemployment, and proportion of households on welfare. Like parental SES, neighborhood affluence was viewed as a formative latent variable with an inter-item consistency of .86. Neighborhood affluence scores were calculated as the standardized sum of standardized indicator scores. The rank-order stability of neighborhood affluence was .83 from the first to the second year and .57 from the first wave to year 10. Neighborhood residential stability (further abbreviated as neighborhood stability) was operationalized as neighborhoods’ standardized proportion of households living in the same house for more than five years. The rank-order stability of neighborhood stability was .80 from the first to the second year and .35 from the first wave to year 10.

#### Parenting

We distinguished between family stress and three core parenting practices related to delinquency among youths: parental knowledge, parental involvement, and parental reinforcement (e.g., [19]). Family stress referred to caretakers’ perceptions of their stress levels and ability to handle problems. The construct was measured using 14 items with a 3-point Likert-scale. An example of an item is “You felt unable to control the important things in your life.” The items were used as a scale that demonstrated good reliability properties in our sample, ranging from α = .83 to .88 across waves.

Parental knowledge refers to the extent of caretakers’ knowledge of youth’ activities. The construct was measured using four items from both the youth and the primary caretaker with a 3-point Likert-scale. An example of a youth-report item is “Do your parent(s) know who you are with when you are away from home?” The items were used as a scale that demonstrated adequate reliability properties in our sample, ranging from α = .63 to .64 across waves for caretakers and from α = .67 to .73 for youths. Parental involvement refers to how much time youths spend with their caretakers. It was assessed using a caretaker report of the number of hours per week spent together. Parental reinforcement refers to the frequency of caretakers’ positive behaviors towards youths, such as giving special privileges or compliments. The construct was measured using nine items from the caretaker and seven items from the youth on a 3-point Likert scale. An example of an item is “When your son did something that you liked or approved of, how often did you give him a hug, pat on the back, or a kiss for it.” The items were used as a scale that demonstrated good reliability properties in our sample, ranging from α = .77 to .86 across waves for caretakers and from α = .71 to .86 for youths. Family stress and the three parenting constructs were standardized. Bivariate correlations between all variables in this study are listed in Table 2.

**Table 2:** Bivariate Correlations (Pearson’s R). *Note*. Estimates across observations with standard errors adjusted for clustering within respondents. The first three waves are excluded due to a low prevalence of delinquency. Correlations between different types of delinquency are the square root of a logistic regression analysis’ pseudo R^2^ (row variable regressed on column variable).

Variable	2	3	4	5	6	7	8	9	10
1. Family SES	.44***	.15***	-.15***	.20***	-.03	.01	-.09***	-.13***	-.14***
2. Neighborhood Affluence		.29***	-.06*	.16***	-.06*	-.04	-.03	-.10***	-.06*
3. Neighborhood Stability			-.02	.10***	-.03	-.06	-.07**	-.09***	-.09***
4. Family Stress				-.26***	-.11***	-.17***	.13***	.12***	.09***
5. Parental Knowledge					.21***	.37***	-.25***	-.15***	-.18***
6. Parental Involvement						.22***	-.11***	-.07**	-.08***
7. Parental Reinforcement							-.13***	-.04	-.07**
8. Minor Delinquency								.23***	.39***
9. Moderate Delinquency									.31***
10. Serious Delinquency									

* p < .05.

** p < .01.

*** p < .001.

### Strategy of Analysis

#### Statistical method

This study used logistic regression models with maximum likelihood estimation to predict the occurrence of delinquency. For each type of delinquency, we separately carried out two types of analysis. First, we used random effects logistic regression analyses with time-constant predictors (based on respondents’ over-time mean) to assess *between-individual associations*. Second, we used fixed effects logistic regression analyses [45] to assess *within-individual associations*. A negative association between SES and delinquency in a fixed effects model implies that respondents were more likely to offend during years in which their parents’ SES was lower than during years in which their parents’ SES was higher relative to their own average level of SES across all time points. By estimating the effect of over-time changes, fixed effects models control for all possible time-constant factors such as ethnicity or a genetic propensity to commit crime. For the mediation analysis, we added comparable models with continuous variables (the mediators) as outcome variable. These models were estimated using ordinary least squares with heteroscedasticity and cluster (within respondent) robust standard errors [46].

#### Model specification

Analyses were carried out both between and within individuals. For each type of delinquency, we first tested our hypothesis regarding the effect of SES using a model with only SES as predictor. Second, we assessed the effect of the mediators by adding these to the model. To obtain a more accurate estimate of total SES and neighborhoods effects, we thirdly specified a model that added an interactional effect between SES and neighborhood affluence using a multiplicative term of both centered variables. In the within-individual models, this interaction was cross-level with neighborhood quality at the between-level and SES at the within-level. Previous research indicates that neighborhood effects may be more accurately depicted if they are allowed to vary over different levels of risk factors such as SES [47–48]. Prior to analyses, we verified the assumption that SES displays no significant interaction effects with age (either linear or quadratic) on delinquency.

#### Mediation

For each hypothesized mediator, we assessed the percentage of the total SES effect on delinquency that was mediated. For this purpose, we proceeded in two steps [49]. First, we used the previously described model with SES and all mediators as predictors and delinquency as the dependent variable. Second, we specified models with SES as predictor and each separate mediator as the outcome variable. For each mediator, we calculated indirect effects by multiplying its effect on delinquency from the first step with its corresponding SES effect from the second step. We then transformed the indirect effects to percentages of the total SES effect (the sum of all indirect effects and the direct SES effect). These percentages indicate the unique contribution of each mediator in explaining the effect of SES, after controlling for all other mediators. Finally, we assessed the statistical significance of the mediational effect using a Sobel test [50].

#### Control variables

A fixed set of control variables was included in all analyses consisting of age dummies, a wave dummy, ethnicity (only necessary in between-individual models), and single parenthood. An age dummy was included for each (full year) age to control for the differential probability of offending at different ages (i.e., the age crime curve) and for mean level increases in SES during the course of the study. A dummy indicating the first three waves was included because delinquency was measured using a somewhat different set of instruments at these waves. Ethnicity and single parenthood were controlled for because they could plausibly affect both SES and delinquency. Specifically, the single parenthood variable controlled for the possibility that parental divorces affected both SES and delinquency over time.

#### Missing values

We assessed the amount of missing values across all 5030 (503 respondents at 10 waves) observations. The percentage missing for each variable was as follows: Minor delinquency 0.1%, moderate delinquency 0.1%, serious delinquency 0.1%, family SES 24.7%, neighborhood affluence 3.7%, neighborhood stability 3.7%, parental knowledge 8.0%, parental involvement 8.5%, parental reinforcement 7.8%, single caretaker 7.7%. Due to the used estimation method, all respondents who had no missing values on at least one wave could be included in the analyses. For every respondent, only waves with complete data were used.

## Results

### SES and Delinquency

**Fig 1:**
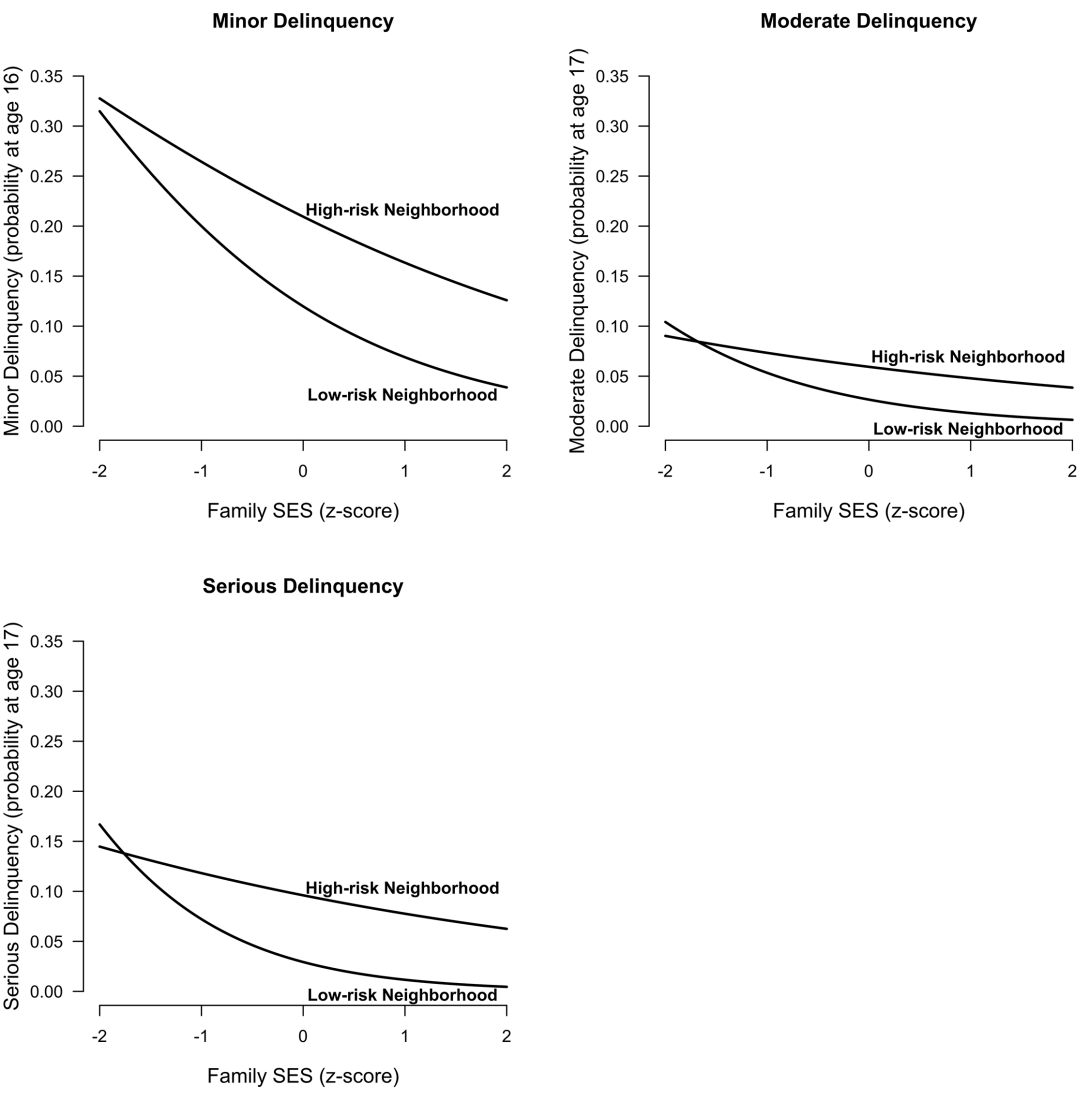
Between Individuals: Probability of Offending as a Function of Family SES by Neighborhood Quality. Estimates obtained from between-individual model 3 (z-score of 1 or -1 on neighborhood affluence and stability).

To examine the association between SES and delinquency, we specified models with only SES as predictor of interest. All analyses in this study furthermore included the aforementioned control variables. Between individuals, SES displayed a negative effect on all three types of delinquency. As hypothesized (H1), low-SES youths were more likely to offend than high-SES youths. Based on the model’s prediction, low-SES youths (z-score = -1) were about two times more likely to commit minor delinquency, two and a half times more likely to commit moderate delinquency, and three times more likely to commit serious delinquency than high-SES youths (z-score = +1; depicted in Fig 1).

**Fig 2:**
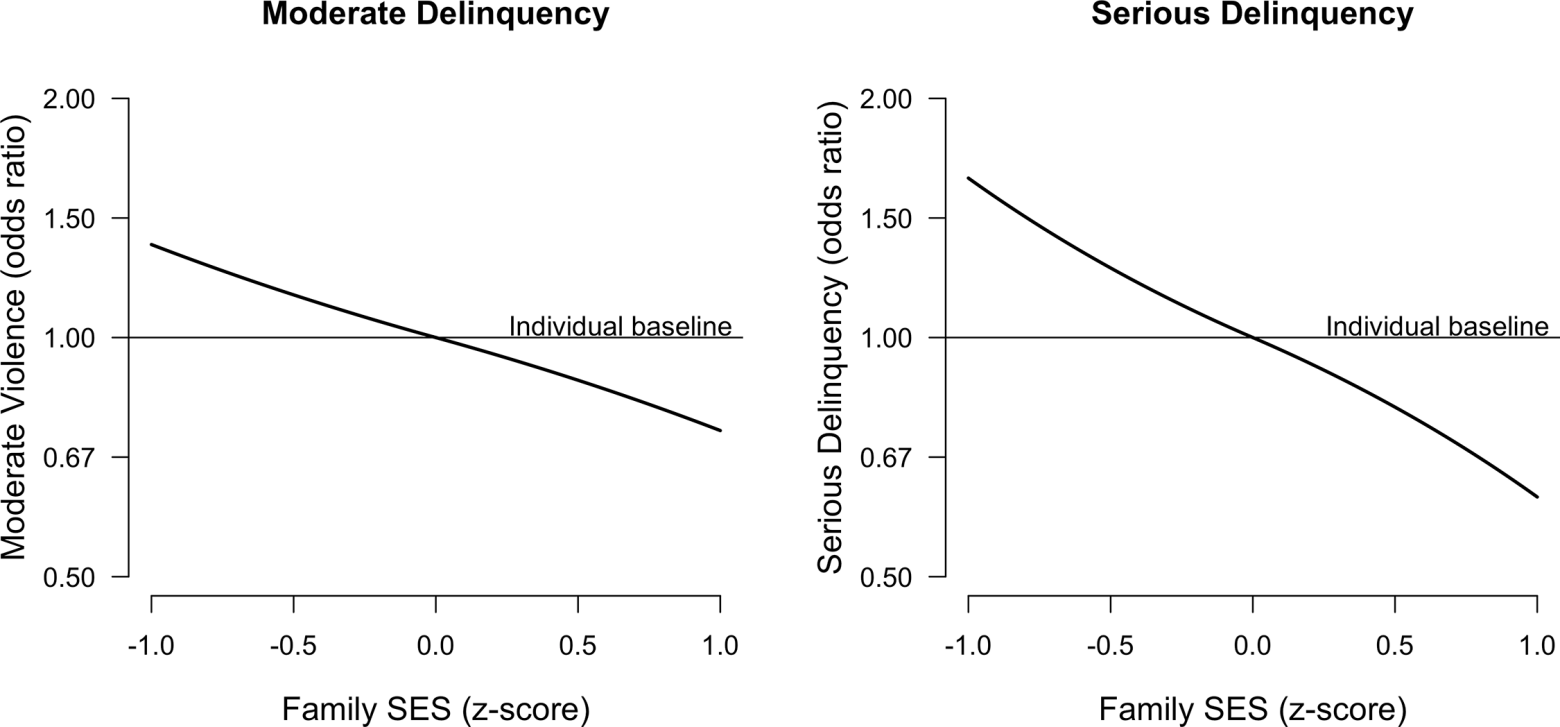
Within Individuals: Relative Odds of Offending as a Function of Family SES. The graph shows that if family SES is at a respondent’s individual (across waves) mean, odds of offending are at the respondent’s individual baseline. As family SES changes, odds of offending are multiplied with the score on the y-axis.

Within individuals, SES revealed a negative effect on moderate and serious delinquency, but not on minor delinquency. As hypothesized (H1), youths were more likely to commit moderate and serious delinquency during years in which their parents’ SES was lower than during years in which their parents’ SES was higher. In other words, youths changed their delinquent behavior when their family SES changed. In years that their parents’ SES was lower (z-score = -1), youths had about one and a half times higher odds of offending than in years that their parents’ SES was at their personal (across years) average (depicted in Fig 2). Regression models are displayed in Table 3, Table 4 and Table 5 (predictors of interest), supplemented by control variables in (Appendix A in S1 File).

**Table 3:** Regression Models for Minor Delinquency. *Note*. Odds ratios with standard errors in parentheses. Control variables omitted from table: single caretaker, ethnicity, and age dummies.

	Between			Within		
Predictor	(1)	(2)	(3)	(1)	(2)	(3)
SES	0.59 (0.06)***	0.74 (0.08)**	0.63 (0.08)***	1.14 (0.15)	1.13 (0.15)	1.19 (0.16)
Neighborhood						
Affluence		1.05 (0.13)	0.90 (0.13)		0.92 (0.11)	0.95 (0.11)
Stability		0.81 (0.08)*	0.80 (0.08)*		0.94 (0.08)	0.95 (0.08)
SES*Affluence			0.86 (0.10)			1.33 (0.17)*
Stress and Parenting						
Family Stress		1.07 (0.10)			1.12 (0.10)	
Knowledge		0.48 (0.05)***			0.79 (0.06)**	
Involvement		0.89 (0.08)			0.82 (0.06)*	
Reinforcement		1.09 (0.11)			0.86 (0.08)	

* p < .05.

** p < .01.

*** p < .001.

**Table 4:** Regression Models for Moderate Delinquency. *Note*. Odds ratios with standard errors in parentheses. Control variables omitted from table: single caretaker, ethnicity, age dummies, and wave dummy.

	Between			Within		
Predictor	(1)	(2)	(3)	(1)	(2)	(3)
SES	0.56 (0.08)***	0.69 (0.10)*	0.62 (0.09)**	0.72 (0.11)*	0.71 (0.12)*	0.66 (0.11)*
Neighborhood						
Affluence		0.85 (0.13)	0.75 (0.13)		0.85 (0.12)	0.85 (0.11)
Stability		0.88 (0.12)	0.87 (0.12)		0.88 (0.09)	0.88 (0.09)
SES*Affluence			0.78 (0.11)			0.82 (0.14)
Stress and Parenting						
Family Stress		1.42 (0.18)**			1.11 (0.13)	
Knowledge		0.76 (0.11)			0.83 (0.08)	
Involvement		0.65 (0.08)***			1.08 (0.11)	
Reinforcement		1.13 (0.14)			0.93 (0.10)	

* p < .05.

** p < .01.

*** p < .001.

**Table 5:** Regression Models for Serious Delinquency. *Note*. Odds ratios with standard errors in parentheses. Control variables omitted from table: single caretaker, ethnicity, age dummies, and wave dummy.

	Between			Within		
Predictor	(1)	(2)	(3)	(1)	(2)	(3)
SES	0.48 (0.07)***	0.61 (0.10)**	0.55 (0.09)***	0.60 (0.11)***	0.53 (0.10)***	0.49 (0.10)***
Neighborhood						
Affluence		0.86 (0.14)	0.68 (0.13)*		1.21 (0.20)	1.15 (0.18)
Stability		0.79 (0.12)	0.78 (0.01)		0.84 (0.10)	0.81 (0.10)
SES*Affluence			0.70 (0.11)*			0.87 (0.15)
Stress and Parenting						
Family Stress		1.16 (0.16)			1.14 (0.14)	
Knowledge		0.57 (0.09)***			0.89 (0.10)	
Involvement		0.71 (0.10)*			0.94 (0.10)	
Reinforcement		1.16 (0.16)			0.98 (0.12)	

* p < .05.

** p < .01.

*** p < .001.

### Mediation Analysis

#### Mediators and delinquency

To examine the associations of neighborhood quality and parenting with delinquency, we specified models with SES and these hypothesized mediators as predictors. Between individuals, neighborhood stability displayed a negative effect on minor delinquency. Youths in more stable neighborhoods were less likely to offend. Parental knowledge had a negative effect on all three types of delinquency. Youths with parents who knew less about their activities were more likely commit delinquency. Parental involvement revealed a negative effect on moderate and serious delinquency. Youths who spent less time with their parents were more likely to offend. Family stress displayed a positive effect on moderate delinquency. Youths with parents who experienced higher stress levels were more likely to commit delinquency. Within individuals, parental knowledge and involvement displayed a negative effect on minor delinquency. Youths were more likely to offend during years in which their parents knew less about their activities and spent less time with them. All other associations between hypothesized mediators and delinquency were non-significant. Overall, our hypotheses that a lower neighborhood quality (H2) and less positive parenting (H3) would be related to a higher probability of offending was only partially supported.

#### SES and mediators

To assess the associations between SES and the hypothesized mediators, we specified separate models with each of these mediators as outcome variable and SES as predictor. Between individuals, SES had a positive effect on neighborhood affluence and parental knowledge and a negative effect on family stress. Youths with a higher SES lived in more affluent neighborhoods, and had parents who experienced less stress and had more knowledge about their activities. Within individuals, SES had a positive effect on neighborhood affluence. Youths lived in more affluent neighborhoods during years in which their parents’ SES was higher than during years in which their parents’ SES was lower. All other associations between SES and the mediators (displayed in Table 6) were non-significant.

**Table 6:** Subsequent Regression Models for the Effect of Family SES on Neighborhood Quality and Parenting. *Note*. Unstandardized regression coefficient with standard errors in parentheses. Control variables omitted from table: single caretaker, ethnicity, age dummies, and wave dummy.

Outcome	Between	Within
Neighborhood		
Affluence	0.28 (0.03)***	0.16 (0.03)***
Stability	0.02 (0.04)	0.01 (0.04)
Stress and Parenting		
Family Stress	-0.17 (0.04)***	-0.06 (0.04)
Knowledge	0.17 (0.03)***	0.03 (0.04)
Involvement	-0.01 (0.03)	-0.03 (0.04)
Reinforcement	0.05 (0.03)	0.03 (0.04)

*** p < .001.

#### Mediational effects

For each hypothesized mediator, we assessed what percentage of the total SES effect it mediated and whether this mediational effect was significant. Minor delinquency was excluded from the within-individual mediation analysis because no within-individual SES effect was found to be mediated. Between individuals, parental knowledge displayed a mediational effect for minor and serious delinquency. Family stress revealed a mediational effect for moderate delinquency. All other hypothesized mediational effects were non-significant.

Overall, we found no support for our hypothesis (H4) that neighborhood quality mediates the association between SES and delinquency. Our hypothesis (H5) that parenting mediates this association was supported only between individuals and only for parental knowledge and (for moderate delinquency) family stress. No evidence was found for mediation of the within-individual association between SES and delinquency. Results of the mediation analysis are displayed in Table 7.

**Table 7:** Percentage of SES Effect Accounted for by Mediator. *Note*. Significance tested using Sobel test [50].

Outcome	Between	Within
Minor Delinquency		
Neighborhood Affluence	-2.9	
Neighborhood Stability	0.8	
Family Stress	2.6	
Parental Knowledge	29.3***	
Parental Involvement	-0.4	
Parental Reinforcement	-0.9	
Moderate Delinquency		
Neighborhood Affluence	8.8	6.8
Neighborhood Stability	0.4	0.3
Family Stress	11.5*	1.6
Parental Knowledge	9.2	1.2
Parental Involvement	-1.2	0.6
Parental Reinforcement	-1.1	0.6
Serious Delinquency		
Neighborhood Affluence	6.1	-4.9
Neighborhood Stability	0.6	0.2
Family Stress	3.9	1.3
Parental Knowledge	14.8**	0.5
Parental Involvement	-0.7	-0.3
Parental Reinforcement	-1.1	0.1

* p < .05.

** p < .01.

*** p < .001.

## Discussion

This study first replicated the well-established finding that youths with a lower SES are more likely to offend than others with a higher SES. However, this study’s key finding is that SES is also related to delinquency within individuals. Research indicates that it is very common for American youths to experience poverty during some years, but not during others [5]. This study revealed that youths were indeed more likely to commit moderate delinquency and serious delinquency during those years in which their parents’ SES is lower than during those years in which their parents’ SES is higher. Contrary to our expectations, we found no evidence that this within-individual association was accounted for by families moving to different neighborhoods or by changes in parenting.

### Socioeconomic Status

Many theoretical accounts have proposed that a low SES contributes to delinquency, either through economic deprivation (e.g., [7]), poor neighbor quality (e.g., [10]), or a lack of positive parenting (e.g., [17]). Consistent with all these theories, this study’s findings strongly emphasize the importance of SES as a correlate of juvenile delinquency. First, this study revealed a particularly strong between-individual association with SES for all types of delinquency; especially in combination with neighborhood quality. In comparison with high-SES youths (z-score = +1) in low-risk neighborhoods (z-score = +1), low-SES youths (z-score = -1) in high-risk neighborhoods (z-score = -1) were about four times more likely to commit minor delinquency, five and a half times more likely to commit moderate delinquency, and ten times more likely to commit serious delinquency (Fig 1). These associations are stronger than those found in many other studies [1; 51]. This difference may potentially be explained by the fact that this study combined four different aspects of SES, whereas many other studies included only one or two. Furthermore, SES effects may have been larger in this study because we distinguished between different types of delinquency [1]. Previous research indicates that SES may primarily be related to more severe types of offending [42]. Although we made no explicit comparison, this study revealed a similar pattern.

Second, this study emphasized the relevance of SES by revealing that it is related to delinquency not only between, but also within individuals. The same youths were more likely to offend during years in which their parents’ SES was lower than during years in which their parents’ SES was higher. In other words, youths changed their delinquent behavior as their parents’ SES changed. This within-individual association with SES was found for moderate delinquency and for serious delinquency, but not for minor delinquency. This discrepancy may result from the fact that SES is related primarily to more serious types of delinquency, thereby making it harder to observe a within-individual association with less severe offenses.

### Neighborhoods

Social disorganization theory (e.g., [10]) proposes that poor and disorganized neighborhoods facilitate delinquency due to a lack of social capital and collective supervision. Although we indeed found a negative between-individual association of neighborhood residential stability with minor delinquency, all other neighborhood effects were non-significant in the multivariate models. Therefore, we found no support for our hypothesis that within-individual changes in delinquency might occur as youths move from one neighborhood to another. Also, we found no support for our hypothesis that the association between SES and delinquency is partly accounted for by neighborhoods.

### Parenting

Control theory (e.g., [17]) argues that a positive parent-child relationship can constitute a social bond that prevents delinquency. Our between-individual findings supported this notion by demonstrating that youths were less likely to offend if their parents knew more about their activities and spent more time with them. We furthermore expected within-individual changes in parenting to be related to changes in delinquency. Support for this hypothesis was limited to minor delinquency: Youths were more likely to commit minor delinquency during years in which they spent less time with their parents and during years in which their parents knew less about their activities. Drawing from the family stress model (e.g., [15]), we finally hypothesized that the association between SES and delinquency would partly be accounted for by parenting. Support for this hypothesis was limited. Between individuals, parental knowledge displayed a mediating role. However, we found no evidence that the within-individual association between SES and delinquency was accounted for by changes in parenting. In fact, we found no within-individual association with SES for any of the four parenting characteristics.

### Theoretical Implications

This study aimed to contribute to the theoretical debate on the association between SES and delinquency. Social causation accounts (e.g., [7; 10; 17]) view SES not only as a correlate but also as a cause of delinquency. This study’s finding that youths change their delinquent behavior as their parents’ SES changes lends support for these accounts. The models used to estimate this within-individual association controlled for all time-constant factors as well as some additional control variables, hence lending stronger support for causal inferences than cross-sectional research. Since this is still a correlational study, caution for causal inferences is warranted as unmeasured time-varying confounding variables may account for the observed findings. This study’s findings do however suggest that causal findings from experimental research on SES and externalizing behavior [27] may translate to delinquency. This study therefore challenged claims that the association between SES and delinquency is either spurious or originating from earlier life-phases (e.g., [34–35; 37]). This study’s findings do not refute such theories, but they do suggest that these explanations do not account for the complete association between SES and delinquency.

Though this study established a within-individual association between SES and delinquency, it remains an open question what mechanism may explain this relation. It may be that neighborhoods and parenting do in fact mediate this association, but that this study failed to detect this mediational effect. Alternatively, the within-individual association between SES and delinquency may result because youth begin to view criminal behavior as one of the best ways to obtain resources when their families have low economic recourses, as suggested by strain and rational choice theories.

### Strengths and Limitations

This study’s primary strength was that it provided us with a unique opportunity to assess a within-individual association between SES and offending because of its long time span, high prevalence of delinquency, and substantial within-individual variability in SES. Furthermore, the strong representation of low-SES youths in our sample allowed us to study a group that may be particularly sensitive to changes in SES. Another strength of this study was its comprehensive operationalization of SES. Combining four aspects of SES into a single composite allowed us to study their combined effect, hence maximizing power. A drawback of this strategy is that we could not distinguish the effects of individual components [52]. However, we reasoned that over-time changes in different aspects of SES would interrelate too strongly (e.g., losing a job and income) to make a meaningful distinction.

A limitation of this study was that we lacked sufficient data to study subjective economic deprivation as an explanatory mechanism. Another limitation was that this study did not assess bidirectional associations. Including such bidirectional effects would have come at a price for power and parsimony. We reasoned that a reverse effect of youths’ delinquency on family SES is not plausible enough to justify this tradeoff. For parenting, it contrarily seems very likely that the associations we found with delinquency are partly bidirectional [30; 53–54]. Disentangling this bidirectional association was however not an objective of this study. A third limitation of this study was the relatively high amount of missing values on family SES (24.7%), which could have created sample selection bias if the effect observed among included cases differed from the effect among excluded cases.

## Conclusion

In sum, this study’s key finding was that youths were more likely to offend during years in which their parents’ SES was lower than during years in which their parents’ SES was higher. These findings indicate that changes in SES, like parental job losses or promotions, are related to changes in youths’ delinquent behavior. Since within-individual models provide a stricter test of causality than between-individual analyses, this study supports claims that impacting familial SES may have a direct effect on youths’ delinquency. While the explanatory mechanism for this within-individual association remains an open question for future research, these findings therefore suggest that programs designed to improve familial SES may be particularly effective at promoting desistance from crime among youth during the adolescent years.

## Supporting Information

S1 File Effects of Control Variables in Different Models (Appendix A in S1 File). (DOCX)
