# Supplementary material for: Moving in and out of Poverty: The Within-Individual Association between Socioeconomic Status and Juvenile Delinquency
Source: PLoS One. 2015 Nov 17;10(11):e0136461. doi: 10.1371/journal.pone.0136461 (PMC4648521; doi:10.1371/journal.pone.0136461)
Supplement: S1 File — (DOCX) [file pone.0136461.s001.docx]

**Appendix A. Effects of Control Variables in Different Models.**

|  | Between | | |  | Within | | | |
| --- | --- | --- | --- | --- | --- | --- | --- | --- |
| Predictor by Outcome | (1) | (2) | (3) |  | | (1) | (2) | (3) |
| Minor Delinquency |  |  |  |  | |  |  |  |
| Single Caretaker | 1.03 (0.15) | 0.99 (0.14) | 1.04 (0.15) |  | | 1.04 (0.17) | 1.02 (0.17) | 1.02 (0.17) |
| African-American | 1.20 (0.25) | 0.83 (0.20) | 0.94 (0.24) |  | |  |  |  |
| Moderate Delinquency |  |  |  |  | |  |  |  |
| Single Caretaker | 0.84 (0.15) | 0.82 (0.15) | 0.86 (0.15) |  | | 0.71 (0.14) | 0.72 (0.15) | 0.74 (0.15) |
| African-American | 2.55 (0.65)*** | 1.78 (0.56) | 1.82 (0.57) |  | |  |  |  |
| Serious Delinquency |  |  |  |  | |  |  |  |
| Single Caretaker | 1.13 (0.22) | 1.14 (0.22) | 1.15 (0.22) |  | | 0.99 (0.22) | 1.07 (0.25) | 1.03 (0.24) |
| African-American | 1.34 (0.37) | 0.81 (0.27) | 0.82 (0.28) |  | |  |  |  |

*Note.* Odds ratios with standard errors in parentheses. Control variables omitted from table: age dummies and wave dummy. The ethnicity variable was omitted from the within models because it has no over-time variability. *** p < .001.
